# ‘The people in every village they visited were so often mourning the dead’

**DOI:** 10.1098/rstb.2008.4018

**Published:** 2008-11-27

**Authors:** Kainamba Mabage

**Affiliations:** Waisa Village, c/o Papua New Guinea Institute of Medical ResearchPO Box 60, Goroka, EHP 441, Papua New Guinea

I was still young and unmarried when Michael Alpers came into Waisa village in 1962 to live there and do research on kuru, which was taking the lives of the people at an alarming rate. I was among the young girls who cleaned up the land to build his house at Yagoenti. My late husband Mr Mabage was a single young man when Michael employed him. He helped in the house and whenever Michael wanted to go out on field patrol he went with him. He was a very handy man in doing fieldwork. He was also asked to go independently to check on kuru patients and report back whenever the symptoms of kuru had changed in the patients.

When the medical scientists ran out of food they survived on local vegetables provided by staff members and village people. It was not an easy job in the early days of kuru research, when the people in every village they visited were so often mourning the dead. The staff were kept busy all the time collecting samples and helping the relatives in the funerals of patients. When the research officers left the country my late husband was retrenched and we lived a happy life in Waisa until he died of kuru in 1980.

